# Umatilla Virus Genome Sequencing and Phylogenetic Analysis: Identification of Stretch Lagoon Orbivirus as a New Member of the *Umatilla virus* Species

**DOI:** 10.1371/journal.pone.0023605

**Published:** 2011-08-29

**Authors:** Manjunatha N. Belaganahalli, Sushila Maan, Narender S. Maan, Robert Tesh, Houssam Attoui, Peter P. C. Mertens

**Affiliations:** 1 Vector-borne Diseases Programme, Institute for Animal Health, Surrey, United Kingdom; 2 Department of Pathology, University of Texas Medical Branch, Galveston, Texas, United States of America; Veterinary Laboratories Agency, United Kingdom

## Abstract

The genus *Orbivirus*, family *Reoviridae*, includes 22 species of viruses with genomes composed of ten segments of linear dsRNA that are transmitted between their vertebrate hosts by insects or ticks, or with no identified vectors. Full-genome sequence data are available for representative isolates of the insect borne mammalian orbiviruses (including bluetongue virus), as well as a tick borne avian orbivirus (Great Island virus). However, no sequence data are as yet available for the mosquito borne avian orbiviruses.

We report full-length, whole-genome sequence data for Umatilla virus (UMAV), a mosquito borne avian orbivirus from the USA, which belongs to the species *Umatilla virus*. Comparisons of conserved genome segments 1, 2 and 8 (Seg-1, Seg-2 and Seg-8) - encoding the polymerase-VP1, sub-core ‘T2’ protein and core-surface ‘T13’ protein, respectively, show that UMAV groups with the mosquito transmitted mammalian orbiviruses. The highest levels of sequence identity were detected between UMAV and Stretch Lagoon orbivirus (SLOV) from Australia, showing that they belong to the same virus species (with nt/aa identity of 76.04%/88.07% and 77.96%/95.36% in the polymerase and T2 genes and protein, respectively). The data presented here has assisted in identifying the SLOV as a member of the Umatilla serogroup. This sequence data reported here will also facilitate identification of new isolates, and epidemiological studies of viruses belonging to the species *Umatilla virus*.

## Introduction

The genus *Orbivirus* is the largest of 15 genera within the family *Reoviridae* and currently contains 22 recognized virus species, as well as 13 unclassified or ‘unassigned’ viruses, some of which may represent additional species [1], [2], [3], [4], [5]. Many orbiviruses are transmitted by ticks or haematophagus insect-vectors (*Culicoides*, mosquitoes and sand flies), with a wide host range that can collectively include cattle, goats and sheep, wild ruminants, equids, camelids, marsupials, sloths, bats, birds, large canine and feline carnivores, and humans. Members of the different *Orbivirus* species that are currently recognised, were initially distinguished by a lack of cross-reaction in serological assays, including agarose gel immunodifusion, complement fixation and (more recently) enzyme linked immunosorbent assays (ELISA)[2].

The orbivirus genome consists of 10 separate pieces of linear double stranded RNA (dsRNA), most of which code for a single viral protein. In the case of bluetongue virus (BTV - the *Orbivirus* type-species) these include seven structural proteins and four distinct non-structural proteins (Seg-10 encodes two non structural proteins). The components of the virus core-particle and at least two of the non-structural proteins are highly conserved between isolates of the same *Orbivirus* species, showing high level serological cross-reactions in virus species/serogroup-specific assays. The nucleotide and amino acid sequences of these conserved genes and proteins (Polymerase and T2 protein) show variations that primarily reflect the virus serogroup/species. However, within each virus species, they also show variations that reflect the geographic origin of the virus isolate (topotype) [6], [7], [8], [9], [10].

In contrast, the orbivirus outer-capsid proteins (VP2 and VP5 of BTV) are more variable and the specificity of their reactions with the neutralising antibodies that are generated during infection of vertebrate hosts can be used to distinguish and identify distinct serotypes within each virus species [11], [12]. As a consequence, the orbivirus outer-capsid proteins (and genes) show sequence variations within each virus species that correlate primarily with the virus serotype [8], [13]. However, they also show lower levels of topotype-specific variation, in a similar manner to the more conserved genes/proteins [13], [14], [15], [16].

Based on these observations, sequencing and comparisons of the conserved and variable orbivirus genome segments can be used to unambiguously assign novel virus isolates to the individual *Orbivirus* species that have already been recognised [15], [17], [18]. These sequences can also be used to design virus-species specific RT-PCR based assays [19], [20], [21].

Multiple BTV serotypes have recently emerged in Europe and the southern USA, along with BTV-2, BTV-7 (and other orbiviruses) in Australia [16], [22], [23], [24]. These events have been linked to climate change, indicating a continuing threat posed by emerging orbiviruses [25], [26]. Two novel serotypes of BTV have also recently been identified (BTV-25 in Switzerland [15] and BTV-26 in Kuwait [17]. In each case these viruses were identified, and assigned to different serotypes, topotypes and even individual virus lineages, by sequencing and phylogenetic comparisons of their genome segments [8], [15], [16].

Sequence data are currently available for the genome of multiple isolates of several different orbivirus species, including BTV, African horse sickness virus (AHSV) (www.reoviridae.org/dsRNA_virus_proteins/orbivirus-accession-numbers.htm), Epizootic haemorrhagic disease virus (EHDV) [27], Equine encephalosis virus (EEV) (Potgieter *et al.*, - manuscript in preparation), Chuzan virus [28], Peruvian horse sickness virus (PHSV) [29], Yunnan Orbivirus (YUOV) [30], Great Island virus [31] and St'Croix river virus (SCRV) [18]. However, most genome segments of viruses belonging to the thirteen other established *Orbivirus* species remain unsequenced, making molecular diagnosis and unequivocal identification of individual virus strains (e.g. by RT-PCR assays and phylogenetic analyses) more difficult. The recent development of nucleotide sequence based diagnostic systems for BTV, AHSV, EHDV and EEV (including RT-PCR assays, microarrays and phylogenetic analyses) has demonstrated the value and importance of genome sequence data for reliable and well documented reference strains [32]. We have therefore sequenced the entire genome of Umatilla virus which belongs to the *Umatilla species* of the genus *Orbivirus*, adding one more member to the list of fully characterised orbiviruses. As Umatilla virus is the first avian borne orbivirus to be fully sequenced it will inevitably increase our knowledge of the genetic relationships of these viruses and may help us to explore the molecular mechanisms involved in their evolution.

The species *Umatilla virus* contains 4 recognised serotypes: Umatilla (UMAV), Minnal (MINV), Netivot (NETV) and Llano Seco viruses (LLSV) [2]. UMAV was first isolated from a pool of *Culex pipiens* mosquitoes collected on 30^th^ July 1969 in Umatilla County, Oregon, USA. The virus was subsequently also isolated from a sample originally collected from house sparrows (*Passer domesticus*) in Texas in 1967 [1]. Stretch Lagoon orbivirus (SLOV) was isolated in 2002 from pooled *Culex annulirostris* mosquitoes, collected at Stretch Lagoon, near the Wolfe Creek National Park in the Kimberley region of Western Australia [3]. Based on genetic characterization of RNA polymerase and T2 proteins, and comparisons to the limited sequence data available for the members of existing *Orbivirus* species, SLOV was proposed as a novel species within the genus. Six additional isolates of SLOV were made from mosquitoes in different locations in Australia, and serological surveys identified horses, donkeys and goats as vertebrate hosts [3]. Another orbivirus, closely related to SLOV, was also isolated from *Aedes alternans* collected in Sydney (Eastern Australia) [33].

This paper reports full genome sequence-analysis of Umatilla virus (UMAV), isolated from a pool of mosquitoes in North America, and comparisons of its polymerase (Pol), T2-subcore-protein, and T13-core-proteins to different orbiviruses, including Stretch Lagoon orbivirus, indicating that UMAV and SLOV both belong to the species *Umatilla virus*.

## Results

### UMAV electropherotype analysis

**Figure 1:**
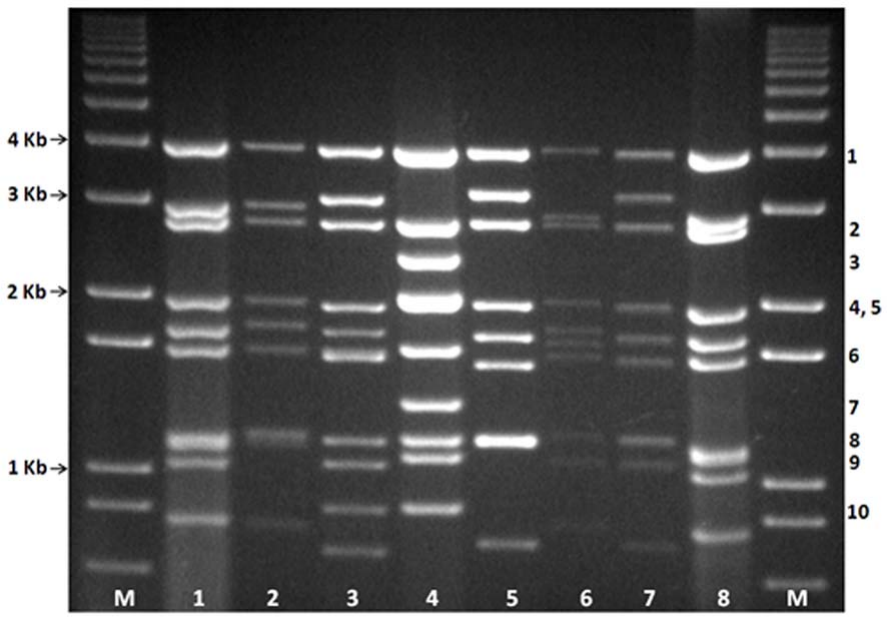
Agarose gel (1%) profile of dsRNA of Umatilla virus along with other orbiviruses. M: 1 Kb DNA Marker, 1: BTV-1 (LIB2007/07); EHDV-6 (USA2006/05); PALV (SUD1983/09); AHSV-1 (RSArah1/03); PHSV (PER1997/01); EEV (RSA1967/02) and BTV-15 (RSArrrr/15) (Figure 1).

The dsRNA of UMAV, purified from infected cell-cultures, was analysed by 1% agarose gel electrophoresis, along with the RNAs of seven other orbiviruses which include: BTV-1 (LIB2007/07); EHDV-6 (USA2006/05); PALV (SUD1983/09); AHSV-1 (RSArah1/03); PHSV (PER1997/01); EEV (RSA1967/02) and BTV-15 (RSArrrr/15) (Figure 1). The majority of the UMAV genome segments migrate separately, with the exception of Seg-4 and Seg-5 which co-migrate in this gel system, generating a ‘1-1-1-2-1-1-2-1’ pattern (Lane 4, Figure 1). In contrast BTV, EHDV, AHSV, EEV and PHSV all show basically similar 3-3-3-1 migration patterns, and PALV show 3-3-4 migration pattern confirming that UMAV belongs to a different species.

### Sequence analysis of the UMAV genome

**Figure 2:**
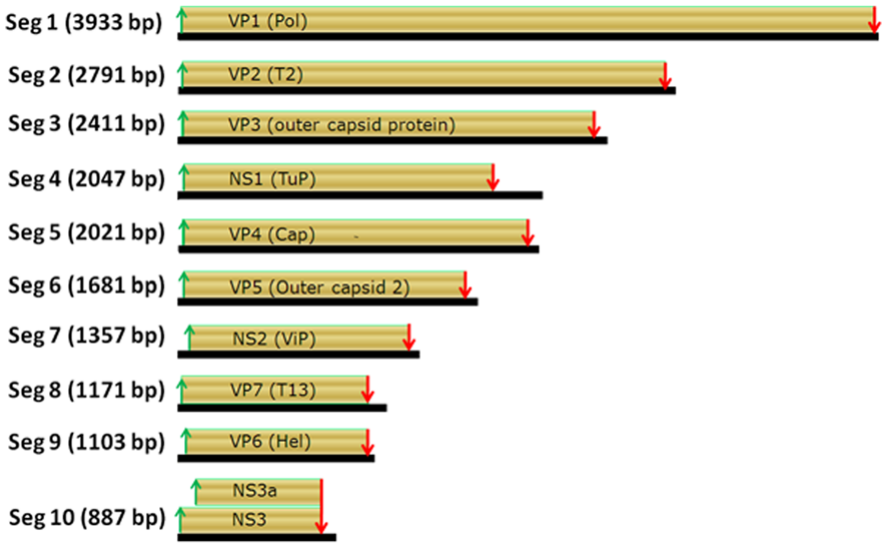
Genome organization of Umatilla virus (UMAV), containing 10 dsRNA segments, each contain an ORF, except Seg-10, which contains two ORFs. The green and red arrows indicates the start and stop codon of the ORF respectively.

Details of size of UMAV genome segments, encoded proteins and properties are given in Table 1. The full genome of the UMAV is 19,402 base pair (bp), with genome segment sizes that range from 3933 to 887 bp, encoding proteins 1299 to 236 aa in length (Table 1; Figure 2). These sequence data have been deposited in the GenBank database (accession numbers HQ842619 to HQ842628 for Seg-1 to Seg-10 respectively). Size differences in the genome segments of UMAV, as compared to other *Culicoides* borne viruses (e.g. BTV) and tick borne viruses (e.g. GIV) are given in Table 2.

**Table 1:** Characteristics of dsRNA genome segments and proteins of Umatilla virus (USA1969/01).

Seg No.	Seg size (bp)	ORFs bp (including stop codon)	Protein nomenclature (protein structure/putative function)	No. of amino acids	Predicted protein molecular mass (KDa)	G+C content (%)	5′ non-coding nucleotide sequence of genome segment (positive strand)	3′ non-coding nucleotide sequence of positive strand (Including stop codon)	Accession Numbers
**1**	3933	13–3912	VP1 (Pol)	1299	148.135	39.05	5′–GUUUUAGUUGCC	**UAG**UCGAGAGGCAACAAAAGAUAC-3′	HQ842619
**2**	2791	19–2736	VP2 (T2)	905	103.654	41.53	5′–GUUUAAGAUGACUCAGCA	**UGA**GCACGAGAAAUCAGCCACCUCGCGUUCCUCCUGGAAGGGGGUCAUCAAAAGAUAC-3′	HQ842620
**3**	2411	19–2344	VP3 (Outer cap)	774	90.151	38.24	5′–GUUUAAAAUCUGAGAGCC	**UAA**UGUAAGCUUGGCACAGCAAUAUGAAGCCCGUGAUCCUAGUCUUAGGUUCGCUCUCAGGUUAAGGAUAC-3′	HQ842621
**4**	2047	30–1772	NS1 (TuP)	580	66.946	40.99	5′–GUUUAAAAACCUAUCGGUGUGCCAGCAUC	**UGA**AAAGAACAUUUUGCAUAACCCACACGAUUAUCAUCAUUUAUCCUUAAUCUUAUUUUUUCGUUUUUAUAAUUAUGUAUUUAGUAAUAGUGUAUGUGUUAGAACAUUUUAUAUCUGUUUUUGUGUAAAAUUAUUAGUAUGUAGAUCAAGAUUAGUGUGUGUACCCGUUUUGUGUGAAAAGCAUUUUAGGCAUAAGAUCCUUAAACCGCUUUGCGAGUGCUCUGUUUGUAUGUAUGUUUCUUGCGUGCAUCUAUAUUAUAGGAUAGGUUUAAGGAUAC-3′	HQ842622
**5**	2021	15–1961	VP4 (Cap)	648	76.048	40.03	5′–GUUUUAGAUGAACG	**UAA**AGCGGACUUGCCUCGGGUCGUCGUGCUCACUUUGGGGAGUGUCAUCGUUCACAAGGAUAC-3′	HQ842623
**6**	1681	29–1618	VP5	529	58.599	42.95	5′–GUUUAAAAUCUUGCAACGGACCGCAGCC	**UAG**UUGCCUCUAUCUUCCUAACGAGCUGGACGUGAAAGUCAAGCGGAGACGCAAGAUUAAGGAUAC-3′	HQ842624
**7**	1357	80–1303	NS2 (ViP)	407	45.883	43.33	5′–GUUUAAAAUCUGACGUACUUUCUGUCACUGGAAGGAGCAUACCAACGAGGAGAAAUCAAGAAAAGUGAAGAGUGAAAAG	**UAA**AGGACGGCUAGUGUUGUAGUGGGAAGCUCACGAUUCGCGUCAGGUUAAGGAUAC- 3′	HQ842625
**8**	1171	18–1073	VP7 (T13)	351	41.554	45.00	5′–GUUUUAAAAGCUACAGC	**UAA**GAGGUAAGGAAGCCCCACACUUUCCCUCUCGCACCCGUGUUGCUCCGCCACGAAGUACCAGGUGUGGGUAUUGAGUGAAUGCUUAGCUUUAAGGAUAC-3′	HQ842626
**9**	1103	43–1068	VP6 (Hel	341	36.471	42.70	5′–GUUUAAAAUCUGAGUAGCUUUUCAUCCUGAGAGAGUCAUUCA	**UAA**CUCAGUGGCCUGGAGCAACUCGGAUCUCAAGAUAC-3′	HQ842627
**10**	887	17–811	NS3	264	29.560	41.71	5′–GUUUAAAAUCUCAAAG	**UAG**ACACUGGAUCGCGGACUGGCACGGUCGAUCUAAGACUACCGGACGUAUGUCGCUACGUCGUUGAGAUUAAGGAUAC-3′	HQ842628
		101–811	NS3a	236	26.422		5′–GUUUAAAAUCUCAAAGAUGUUGGAAGCUGCUGAGCGUGCACAAGAACGGAGUAAAGCAACGGAAUCAGAAACGGUUAGAGAAGACAGUGAUAACGAUUCA		

Pol = RNA polymerase; Outer cap =  outer capsid protein; Cap = capping enzyme (guanylyltransferase); Hel = helicase enzyme; T2 = protein with T = 2 symmetry; T13 = Protein with T = 13 symmetry; ViP = viral inclusion body matrix protein; TuP = tubule protein. Letters in bold are the stop codon.

**Table 2:** Homologous proteins of BTV (*Culicoides* borne), UMAV (mosquito borne) and GIV (tick-borne) orbiviruses.

SegNo	Putative function	BTV genes	*UMAV genes	GIV genes
1	RNA dependent RNA Polymerase (Pol)	Seg-1, VP1(Pol)	Seg-1, VP1(Pol)	Seg-1, VP1(Pol)
2	Outer capsid protein (OCP1)	***Seg-2, VP2(OCP1)***	***Seg-3, VP3(OCP1)***	***Seg-5, VP4(OCP1)***
3	T2, Major subcore protein (T2)	***Seg-3, VP3(T2)***	***Seg-2, VP2(T2)***	***Seg-2, VP2(T2)***
4	Minor core protein - capping enzyme (Cap)	***Seg-4, VP4(Cap)***	***Seg-5, VP4(Cap)***	***Seg-3, VP3(Cap)***
5	Tubule protein (TuP)	***Seg-5, NS1(TuP)***	***Seg-4, NS1(TuP)***	Seg-4, NS1(TuP)
6	Outer capsid protein (OCP2)	Seg-6, VP5(OCP2)	Seg-6, VP5(OCP2)	Seg-6, VP5(OCP2)
7	Major core-surface protein (T13)	***Seg-7, VP7(T13)***	***Seg-8, VP7(T13)***	Seg-8, VP7(T13)
8	Viral inclusion body protein (ViP)	***Seg-8, NS2(ViP)***	***Seg-7, NS2(ViP)***	Seg-7, NS2(ViP)
9	Minor core protein- helicase enzyme (Hel)	Seg-9, VP6(Hel)	Seg-9, VP6(Hel)	Seg-9, VP6(Hel)
10	Virus release protein (VRP)	Seg-10, S3(VRP)	Seg-10, S3(VRP)	Seg-10, S3(VRP)

*Variations in the segments are highlighted in bold italics.

The putative functions of UMAV proteins by comparison to the already established functions of BTV proteins. The functions and abbreviations used as per Mertens et al [2].

The orbivirus genome segments have hexanucleotide termini, which are at least partially conserved (between the different genome-segments) within isolates of each orbivirus species, and to a lesser extent between different species [2] (http://www.reoviridae.org/dsRNA_virus_proteins/CPV-RNA-Termin.htm). Analysis of their terminal non-coding regions (NCRs) showed that the UMAV genome segments share six conserved nucleotides at their 5′ ends, and 7 at their 3′ ends (5′-GUUU^A^/_U_A…..A^G^/_A_GAUAC-3). The first and last 2 nucleotides of each genome segment are inverted complements (Table 1) and are identical to those found in other orbiviruses.

The subcore-shell ‘T2’ protein of UMAV, is encoded by the Seg-2, while the larger outer-capsid and cell-attachment protein (which is also the primary determinant of virus serotype) is translated from Seg-3 (Table 2). This coding assignment is similar to the other mosquito borne orbiviruses [29], [30]. In contrast, the cell-attachment protein is represented by VP2 (encoded by Seg-2) of BTV and the other *Culicoides*-borne orbiviruses [2], and VP4 (encoded by Seg-5) in the tick borne orbiviruses [31].

The UMAV viral inclusion body protein – NS2 (ViP) is encoded by Seg-7 (1357 bp) (which is approximately 200 bp larger than the homologous segment of BTV and other orbiviruses) while the T13 core-surface protein of UMAV (VP7 of BTV) is encoded by Seg-8. In each case these coding assignments are different from those of BTV.

Comparisons with data of other orbiviruses (from GenBank) show that 5.695% of the UMAV genome is non-coding, a percentage similar to that detected in other mosquito borne orbiviruses (5.03% in YUOV and 5.46% in PHSV). However, this is considerably more than the 4.978% (detected in GIV) and 4.47% (in SCRV), both tick-borne viruses, and the 3.5% to 4.1% detected in the *Culicoides* borne orbiviruses.

The UMAV tubule protein ‘NS1(TuP)’ is encoded by Seg-4 (2047 bp), which is 90 to 390 bp larger than the homologous genome segment of the other characterised orbiviruses, although it produces a protein smaller than UMAV VP4, encoded by Seg-5 (2021 bp). This can be explained by the presence of a 275 bp 3′ NCR in Seg-4, the longest NCR that has so far been identified in any of the characterised orbiviruses. Among previously characterised orbiviruses the YUOV has longest 3′ NCR of 202 bp in its NS1 gene (encoding tubular protein). The 5′ NCR of Seg-7 (encoding NS2) and Seg-9 (encoding VP6) of UMAV are longer than the 3′ NCRs (Table 1). In most orbivirus genome segments, the 5′ NCRs are shorter than the 3′ NCRs [2], [8], only Seg-9 of Great Island Virus (GIV) has a longer 5′ NCR (54 bp) than 3′ NCR (39 bp). The significance of a longer 5′ NCRs (in Seg-7 and 9) and the very long 3′ NCR in Seg-4 of UMAV are unknown.

The major ORFs of UMAV Seg-2 (encoding the T2 protein) and Seg-5 (encoding ‘Cap’ protein) both start from the second AUG codon. The first start codon of Seg-2, located at the +8 nt from the upstream end, has a weak Kozak context (**A**AG**AUGA**), [34] and only initiates a small ORF of 87 amino acids ending with an in-frame stop codon at position 271. However, the second start codon, located at +19 position, has a strong Kozak sequence (**G**CA**AUG** G) and initiates a long ORF (19–2736 bp), which encodes the UMAV VP2(T2) protein (906 aa). The first start codon of Seg-5 (encoding VP4(Cap)), also begins at nt +8 but with a strong Kozak context and an in-frame stop codon at nt position +41 from the 5′ end, giving an ORF encoding only 11 aa. Although the second initiation codon (nt 15–1961) has a ‘weak or moderate’ Kozak context, it encodes the 648 aa of the viral capping enzyme ‘VP4 (Cap)’. Several genome segments of other reoviruses and other orbiviruses also have weak or moderate Kozak sequence but still appear to be translated effectively in infected cells [28], [31]. Previous studies have also identified more than one initiation codons near the upstream terminus of BTV Seg-9 and Seg-10, both of which appear to be functional, generating multiple related proteins in infected cells [2], [35], [36], [37], [38]. However, it is unclear if more than one protein is translated from Seg-2 or Seg-5 of UMAV.

The G+C content of the UMAV genome is 41.53%, which falls within the range for mosquito borne orbiviruses, PHSV and YUOV (36.72% and 41.55% respectively). The tick borne orbiviruses previously sequenced have a higher G+C content (57.29% in GIV and 51.93% in SCRV), while the *Culicoides*-borne orbiviruses have an intermediate G+C content of 39.89% (in CHUV) to 45.89% (in EEV).

### Phylogenetic comparisons of orbivirus subcore-shell (T2) proteins

The second largest protein of UMAV ‘VP2’ encoded by Seg-2, was identified as the sub-core shell ‘T2’ protein by BlastX comparisons to homologous proteins of other orbiviruses (VP3 of BTV and VP2 of YUOV, SCRV).

**Figure 3:**
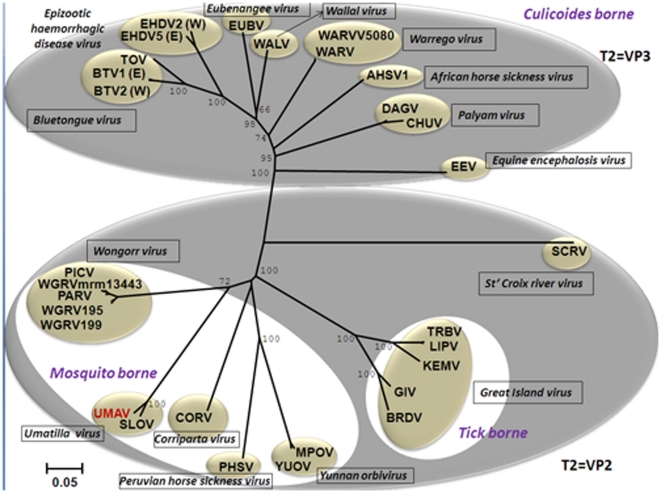
Neighbour-joining tree comparing orbivirus T2 protein sequences. The tree was constructed using distance matrices, generated using the p-distance determination algorithm in MEGA 4.1 (1000 bootstrap replicates) [48]. Since many of the available sequences are incomplete, the analysis is based on partial sequences (aa 393 to 548, numbered with reference to the aa sequence of BTV VP3(T2). The numbers at nodes indicate bootstrap confidence values after 1000 replications. The trees shown in Figures 4 and 5 were drawn using same parameters. The UMAV isolate characterised in this study is shown in red font. Full names of virus isolates and accession numbers of T2 protein sequences used for comparative analysis are listed in (Table S1). ‘(E)’ and ‘(W)’ - indicate eastern and western strains, respectively.

An unrooted neighbour-joining (NJ) tree was constructed for T2 protein sequences of different orbiviruses, listed in the Table S1. These proteins separate into two major clusters (Figure 3): one that includes the *Culicoides* transmitted viruses (including BTV, AHSV, EHDV, *Wallal virus*, *Eubenengee virus*, *Warrego virus* and *Palyam virus*), in which T2 is encoded by Seg-3; while the other cluster includes two sub-groups containing orbiviruses transmitted by ticks (SCRV and Great island virus), or by mosquitoes (Corriparta, Wongorr virus, Peruvian horse sickness virus, Yunnan orbivirus), in both of which T2 is encoded by Seg-2 (Figure 3). These phylogenetic analyses show that VP2 (T2) of UMAV clusters with the mosquito borne orbiviruses.

Orbiviruses share 26–83% amino acid (aa) identity in their T2 protein between different species, with highest identities between BTV and EHDV [31]. Nucleotide (nt) and amino acid (aa) identities in Seg-2 and VP2(T2) of UMAV and SLOV, compared to other orbiviruses are given in Table 3. Briefly, VP2(T2) of UMAV showed aa identity of 23.17% to SCRV; 41.5% to 44.26% to tick-borne orbiviruses; 32.78% to 42.5% to *Culicoides*-borne orbiviruses; and 42.05% to 60.65% to mosquito-borne orbiviruses.

Comparisons of UMAV and Stretch Lagoon orbivirus (SLOV), showed nt/aa identity levels of 77.96%/95.36% in Seg-2/VP2(T2) indicating that SLOV is a member of the *Umatilla virus* species.

The shape parameter ‘alpha’ value (which measures rate variability at different site) obtained from sequences analyses of UMAV and other orbiviruses T2 protein was 1.67. This suggests that different sites are evolving at similar rates within the T2 protein [31].

### Phylogenetic comparisons of orbivirus VP1 (Pol) proteins

**Figure 4:**
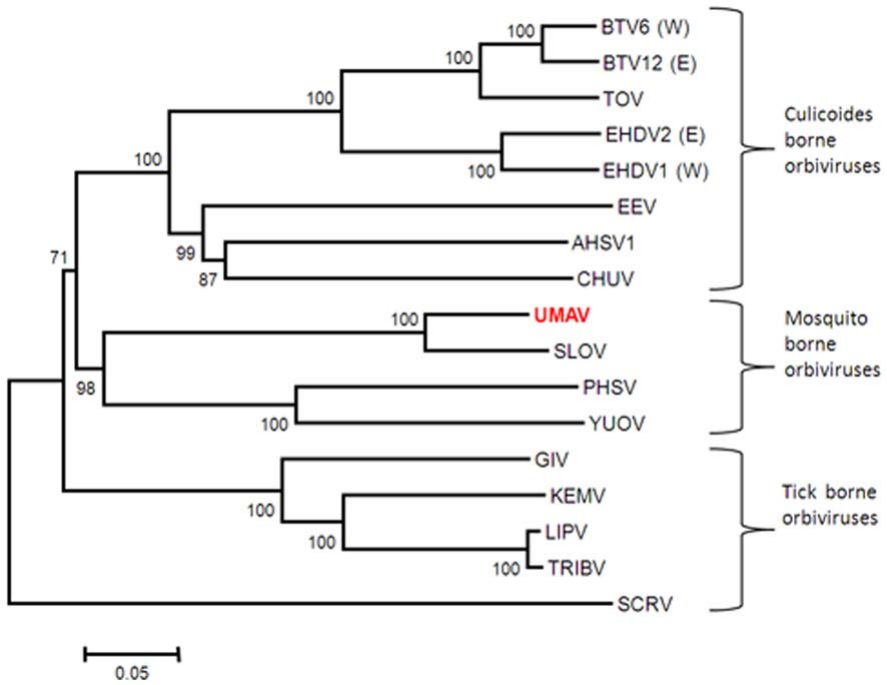
Neighbour-joining tree showing the relationships between amino acid sequences of polymerase protein of different orbiviruses. The UMAV isolate characterised in this study is shown in red font. Full names of virus isolates and accession numbers of polymerase sequences used for comparative analysis are listed in Table S2. ‘(E)’ and ‘(W)’ - indicate eastern and western strains, respectively.

The aa sequence of UMAV VP1 (Pol) and homologous proteins of other *Orbivirus* species, were used to construct a midpoint rooted NJ phylogenetic tree (Figure 4), which clearly shows early separation of the tick borne viruses, followed by the mosquito borne and *Culicoides* borne viruses. This supports the earlier ‘co-speciation’ hypothesis that these viruses have evolved and diverged along with their vector species [31]. The sequences Seg-1 and VP1(Pol) of UMAV and SLOV were compared to those of other orbiviruses (Table S2. The orbivirus polymerase shares 36–73% aa identity between the different *Orbivirus* species that have been characterised [18], [31]. VP1 (Pol) of UMAV shows only 40.06% aa identity to VP1(Pol) of SCRV; 47.74% to 49.22% aa identity to the *Culicoides* borne orbiviruses; 48.59 to 50.78% to the tick borne Great Island viruses; and highest identity levels of 51.16 to 53.13% aa identity to other mosquito borne viruses (Table S2). However, UMAV shares much higher levels of identity (76.04%/88.07% nt/aa identity) with Seg-1/VP1(Pol) of SLOV, confirming that both viruses belong to the same *Umatilla virus* species.

### Phylogenetic comparisons of orbivirus outer-core T13 proteins

**Figure 5:**
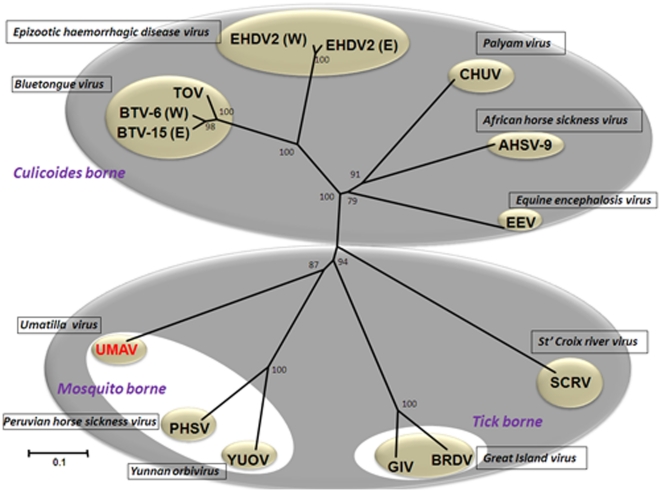
Neighbour-joining tree showing the relationships between amino acid sequences of T13 protein of different orbiviruses. The UMAV isolate characterised in this study is shown in red font. Full names of virus isolates and accession numbers of T13 protein sequences used for comparative analysis are listed in Table S3 ‘(E)’ and ‘(W)’ - indicate eastern and western strains respectively.

The nt sequence of Seg-8 (equivalent to Seg-7 in BTV) and the aa sequence of the T13 outer-core protein of UMAV, were compared to those of other orbiviruses (Table S3). A phylogenetic tree constructed using the T13 aa sequences (Figure 5) shows the same topology as the T2 and polymerase trees. The T13 protein of UMAV shares 22% to 24% aa sequence identity with those of BTV and EHDV and 26.68% identity with tick borne GIV, but shares 30% aa identity with the mosquito borne orbiviruses (Table S3).

## Discussion

Parameters recognised by the International Committee for the Taxonomy of Viruses (ICTV) for the ‘polythetic definition’ of individual *Orbivirus* species include; reassortment of genome segments; genome segment migration patterns during electrophoresis (AGE); conserved terminal nucleotide sequences; serological cross-reactions; comparison of homologous genome segments by sequence analysis or cross-hybridisation; host and vector range and the nature of the clinical signs induced [2]. The majority of the existing *Orbivirus* species were initially recognised as distinct ‘serogroups’. However, reference antisera for these existing species are not widely available, making serological identification of new virus isolates more difficult. Data concerning the potential for reassortment of new orbivirus isolates with members of existing species, would also require access to reference strains and would be laborious to generate.

The genome-segments of viruses within a single *Orbivirus* species, usually show a high level of conservation in their sizes/molecular weights, and consequently have a consistent migration pattern (electropherotype) when analysed by 1% agarose gel electrophoresis (AGE) [2], [13], [39], [40]. The dsRNA profile of UMAV in 1% agarose gel electrophoresis is very different from those of other orbivirus species, with large differences in the sizes and migration pattern of most of the genome segments (Figure 1). This confirms its current classification within a distinct virus species.

Sequence data are becoming increasingly easy to generate and phylogenetic comparison of conserved orbivirus genes (e.g. the T2, Polymerase or T13 protein genes), and can be rapidly compared to existing databases. Consequently they are increasingly being used to identify members of individual virus species [17], [18], [30]. However, this approach inevitably depends on access to sequences for reference strains, data which are not yet available for all of the 22 existing *Orbivirus* species. As part of a programme to create a database representing isolates of each of the *Orbivirus* species, and the currently unassigned orbiviruses, we have sequenced the entire genome of Umatilla virus.

The orbivirus T2 and polymerase proteins that have already been sequenced (Table 3) are relatively highly conserved, with a minimum of 86% and 73% aa identity respectively within a single virus species [31]. These proteins also show 26% to 83%, and 36% to 73% aa identity respectively between different *Orbivirus* species, with highest identities (83% and 73% aa identity in T2 and VP1 (Pol) respectively) between BTV and EHDV [2], [18], [31]. Previous phylogenetic analyses have demonstrated segregation between the T2 = VP2 and T2 = VP3 orbiviruses, reinforcing the hypothesis that the T2 protein is an important evolutionary marker, that can be used to help differentiate/group virus species [18], [30].

**Table 3:** Percent nucleotide (nt) and amino acid (aa) identities of Seg-2 (T2 protein) of UMAV and SLOV with other orbiviruses.

Virus/Nucleotide Accession Number	Percent nucleotide identity		Percent amino acid identity	
	UMAV	SLOV	UMAV	SLOV
SLOV/EU718677	77.96	–	95.36	–
BTV1/E/DQ186822	46.62	46.78	35.82	35.52
BTV2/W/DQ186826	46.54	47.36	35.71	35.19
TOV/W/GQ982523	46.55	47.41	35.82	35.19
EHDV2/W/AM74499	46.14	46.16	35.45	35.38
EHDV5/E/AM74502	46.47	46.78	35.9	35.6
EUBVp/AF530087	50.18	49.3	39.43	39.22
WALVp/AF530084	50.85	50.49	42.5	42.86
WARVp/AF530083	50.97	48.33	42.04	42.45
WARVpV5080/EF21	51.89	49.37	41.56	41.98
AHSV1/AM883166	46.64	46.16	35.81	35.52
CHUV/NC_005989	46.18	46.96	35.85	36.33
DAGVp/AF530085	49.43	47.14	40.01	41.15
EEV/FJ183386	45.22	44.8	32.78	32.81
WGRV195/U56990	59.17	59.67	59.9	59.9
WGRV199/U56991	57.59	59.65	60.65	60.65
WGRVmrm13443/U5	58.22	59.13	58.64	58.88
PARV/U56993	58.54	61.14	60	60
PICV/U56994	57.74	59.93	59.11	59.11
PHSV/NC_007749	52.4	51.03	43.93	43.87
YUOV/NC_007657	50.63	51.06	42.05	43.09
MPOV/EF591620	50.41	49.48	42.16	42.87
CORVp/AF530086	58.37	58.26	57.62	57.32
BRDV/M87875	47.87	48.86	41.5	42.32
GIV/HM543466	48.81	49.4	43.71	44.53
KEMV/HM543482	47.38	49.48	44.15	44.2
LIPV/HM543476	48.9	48.84	44.26	44.64
TRIBV/HM543479	48.5	48.63	44.26	44.64
SCRV/AF133432	37.83	38.34	23.17	22.86

The genetic relationship of UMAV was investigated, showing 23.17% to 60.65% aa identity in the T2 protein and 40.06% to 53.13% aa identity in the viral polymerase to other *Orbivirus* species, confirming its classification as a distinct species. However, UMAV and Stretch Lagoon orbivirus (SLOV) show 95.36% (77.96% nt identity) and 88.07% (76% nt identity) identity in their T2 protein and polymerase aa sequences respectively, indicating that SLOV is a member of the *Umatilla* serogroup and does not represent a new species, as was initially proposed by Cowled et al [3]. Serological studies indicate that SLOV can infect horses, donkeys and goats [3]. However, its close relationship to UMAV, which has been isolated from birds, suggests that both viruses could have a wider host range.

The phylogenetic analysis of conserved T2 genes presented here (Figure 3), also shows phylogenetic segregation between T2 = VP3 (Culicoides borne orbiviruses) and T2 = VP2 (tick and mosquito borne orbiviruses).

The occurrence of closely related viruses in the Americas and Australia, indicates there has been some movement of viruses between these regions, which could be due to movement of hosts or their vectors. Similar movements have also been suggested by the detection of other orbiviruses (PHSV, EHDV) and rhabdoviruses in Australia and North or South America [29], [32], [41], [42].

The orbivirus outer-core ‘T13’ protein (VP7 of BTV) is more conserved than the outer-capsid proteins, and is both immuno-dominant and serogroup/virus-species specific [11], [43]. Consequently the T13 protein represents a primary target for serogroups/species specific serological assays [43]. Sequence comparisons show that VP7(T13) of UMAV exhibits relatively low levels of aa identity with homologous genes of other orbiviruses (30.7% to YUOV, 24% to BTV, 26.86% to GIV and 16% to SCRV), indicating it is not closely related to any of these species [16], [18], [30], [31].

Previous phylogenetic studies of mitochondrial genes indicate that ticks represent ancestors for other arthropods [44]. The analyses presented here, of orbivirus T2, polymerase and T13 proteins, using NJ method and the P-distance algorithm, all show similar grouping of the *Culicoides* borne, mosquito borne and tick borne viruses. In each case UMAV groups with the other mosquito borne viruses (Wongorr virus, PHSV, YUOV and MPOV – Figures 3, 4, 5). The branching of *Culicoides* borne orbiviruses from the mosquito borne viruses, indicates an ancestral relationship. The tick-borne viruses appear to represent ancestors for both the mosquito and *Culicoides* borne orbiviruses as suggested earlier [18], [31]. This further supports the ‘co-speciation hypothesis’ that the orbiviruses have evolved along with their vectors [31].

The data presented here has assisted in identifying the SLOV as a member of the Umatilla serogroup. This will also facilitate the use of sequence data and phylogenetic analyses of novel orbivirus isolates, to identify virus species, serotype and topotype, as well as the arthropod vectors involved in their transmission.

## Materials and Methods

### Virus propagation

UMAV, from the Orbivirus Reference Collection at IAH Pirbright (isolate number USA1969/01), was propagated in BHK-21 cell monolayers, in Dulbecco's minimum essential medium (DMEM) supplemented with antibiotics (100 units/ml penicillin and 100 µg/ml streptomycin) and 2 mM glutamine. Infected cell cultures were incubated at 37°C. The virus was harvested when cell cultures showed widespread (100%) cytopathic effects (CPE) at 48–72 hrs post-infection.

### Agarose Gel Electrophoresis (AGE)

Agarose gels consisted of 1% agarose (Ultra Pure™ Agarose, Invitrogen) in 1× Tris-Acetate EDTA (TAE) buffer with 0.5 µg/ml ethidium bromide. Electrophoresis was carried out at 80 V in 1× TAE running buffer for 16 hr at 4°C in submerged electrophoresis apparatus (SCI-PLAS, UK) and power supply (Biorad). The gels were visualized under UV (Biorad-chemi doc transilluminator) and photographed.

### Preparation of viral dsRNA

Genomic dsRNA of UMAV was extracted from virus infected cell cultures using a guanidinium isothiocyanate extraction procedure. Commercially available TRIzol reagent (Invitrogen) was used, as described by Attoui et al [45]. Briefly, the infected cell pellet was lysed in 1 ml of TRIzol reagent, 0.2 volume of chloroform was added, vortexed and the mixture incubated on ice for 10 min. The supernatant, containing total RNA was separated from the cellular debris and DNA by centrifuging at 10,000×g for 10 min at 4°C. Single stranded RNA (ssRNA) was removed by 2 M LiCl precipitation at 4°C for overnight, followed by centrifugation at 10,000×g for 5 min. An equal volume of isopropanol, containing 750 mM ammonium acetate, was added to the supernatant containing the viral dsRNA, then mixed and allowed to precipitate at −20°C for 2 hours. The dsRNA was pelleted by centrifugation at 10,000×g for 10 min. The pellet was washed with 70% ethanol, air dried and suspended in nuclease free water (NFW). The RNA was either used immediately or stored at −20°C.

### Reverse transcription of dsRNA, and PCR mplification

UMAV genome segments were reverse-transcribed into cDNA using the full-length amplification of cDNAs (FLAC) technique described by Maan et al [46]. Briefly, a 35 base oligonucleotide ‘anchor-primer’, with a phosphorylated 5′ terminus, was ligated to the 3′ ends of the viral dsRNAs using the T4 RNA ligase (New England Biolabs). The ligated dsRNA genome segments were separated by 1% AGE in TAE buffer. The dsRNA bands were excised from the gel in four groups (Seg-1; Seg-2 and 3; Seg-4, 5 and 6; and Seg-7, 8, 9 and 10). The segments were recovered from the gel using silica binding methods (RNaid® kit, MP Biomedicals, Ohio, USA). This is followed by reverse transcription using RT system (Promega). The resulting cDNAs were amplified using complementary primers to anchor primer. PCR amplicons were analyzed by agarose gel electrophoresis. For cloning purposes PCR was done with high fidelity KOD polymerase enzyme (Novagen).

### Cloning and sequencing of cDNA segments

Amplified PCR amplicons were purified and cloned into the Strataclone blunt-end PCR cloning vector ‘pSC-B-amp/kan’ supplied in the StrataClone™ Blunt PCR cloning kit (Stratagene). The recombinant plasmid-vectors were transformed into solopack competent cells supplied along with the kit and recombinant clones identified by blue-white screening. Cloning products (clones) were screened based on touch PCR using M13 universal primers. The clones with desired inserts were identified based on amplicon size in touch PCR and confirmed after sequencing. The plasmids and PCR products were sequenced using an automated ABI 3730 DNA sequencer (Applied Biosystems).

### Sequence analysis and phylogenetic tree construction

‘Raw’ ABI sequence data, was assembled into ‘contigs’ using the SeqManII sequence analysis package (DNAstar version 5). The proteins encoded by the different UMAV genome segments were identified using BlastX (http://blast.ncbi.nlm.nih.gov/Blast.cgi) analysis in GenBank, identifying homologous proteins from the other orbiviruses that have known functional and structural roles. Multiple sequence alignments of consensus sequences were performed using Clustal X (Version 2.0) [47], T-COFFEE, MUSCLE 3.7 (available online at http://www.phylogeny.fr/version2_cgi/one_task.cgi?task_type=muscle) and MAFFT (http://mafft.cbrc.jp/alignment/server/) to ensure correct multiple sequence alignment. Phylogenetic trees were constructed using MEGA 4 software [48] with the p-distance parameter and neighbour joining method [49]. The PAML package was used to calculate shape parameters for gamma distribution analysis [50].

The open reading frame (ORF) in each genome segments was identified and translated to amino acid (aa) sequences using ORF finder in NCBI or EditSeq software (DNAstar version 5.0). The functional and structural role of each encoded protein was identified by comparison to the sequences of other orbiviruses in the international sequence databases (NCBI database). GenBank nucleotide accession numbers of T2, polymerase and T13 protein sequences that were used in phylogenetic analyses are provided in (Table SI).

## Supporting Information

Table S1 **Nucleotide accession numbers for sequences used in phylogenetic analysis.**
(DOC)

Table S2 **Percent identity of UMAV and SLOV Polymerase (Pol) with other orbiviruses.**
(DOC)

Table S3 **Identity levels in the outer-core protein VP7(T13) and gene of UMAV, compared to other orbiviruses.**
(DOC)
