# Supplementary material for: Umatilla Virus Genome Sequencing and Phylogenetic Analysis: Identification of Stretch Lagoon Orbivirus as a New Member of the Umatilla virus Species
Source: PLoS One. 2011 Aug 29;6(8):e23605. doi: 10.1371/journal.pone.0023605 (PMC3163642; doi:10.1371/journal.pone.0023605)
Supplement: Table S2 — Percent identity of UMAV and SLOV Polymerase (Pol) with other orbiviruses. (DOC) [file pone.0023605.s002.doc]

**Table SII**

| **Virus/ Nucleotide Accession Number** | **Percent amino acid (aa) identity** | | **Percent nucleotide (nt) identity** | |
| --- | --- | --- | --- | --- |
|  | **UMAV** | **SLOV** | **UMAV** | **SLOV** |
| SLOV/EU718676 | 88.07 |  | 76.04 |  |
| BTV/W/GQ506536 | 49.22 | 47.35 | 54.79 | 54.17 |
| BTV12/E/GU39065 | 48.83 | 47.27 | 54.22 | 54.06 |
| TOV/GQ982522 | 48.6 | 47.27 | 54.28 | 53.89 |
| EHDV2/E/AM74498 | 47.74 | 46.81 | 55.33 | 54.74 |
| EHDV1/W/AM74497 | 48.36 | 47.43 | 55.18 | 54.25 |
| AHSV1/FJ183364 | 48.72 | 48.25 | 54.8 | 54.06 |
| CHUV/NC_005990 | 48.75 | 48.43 | 55.68 | 53.97 |
| EEV/FJ183384 | 47.83 | 46.66 | 52.77 | 53.54 |
| PHSV/DQ248057 | 53.13 | 51.51 | 57.8 | 56.83 |
| YUOV/AY701509 | 51.16 | 50.23 | 56.78 | 55.74 |
| KEMV/HM543481 | 48.59 | 47.65 | 51.3 | 52.41 |
| LIPV/HM543475 | 50.2 | 49.18 | 51.73 | 52.19 |
| TRIBV/HM543478 | 50.04 | 48.94 | 51.57 | 51.65 |
| GIV/HM543465 | 50.78 | 49.53 | 51.45 | 52.54 |
| SCRV/NC_005997 | 40.06 | 39.83 | 47.07 | 47.18 |
