# Supplementary material for: Umatilla Virus Genome Sequencing and Phylogenetic Analysis: Identification of Stretch Lagoon Orbivirus as a New Member of the Umatilla virus Species
Source: PLoS One. 2011 Aug 29;6(8):e23605. doi: 10.1371/journal.pone.0023605 (PMC3163642; doi:10.1371/journal.pone.0023605)
Supplement: Table S3 — Identity levels in the outer-core protein VP7(T13) and gene of UMAV, compared to other orbiviruses. (DOC) [file pone.0023605.s003.doc]

**Table SIII**:

| **Virus/ Nucleotide Accession Number** | **UMAV** | |
| --- | --- | --- |
|  | **% amino acid (aa) identity** | **% nucleotide (nt) identity** |
| PHSV/NC_007754 | 30.95 | 46.67 |
| YUOV/NC_007663 | 30.77 | 44.71 |
| BRDV/M87876 | 21.84 | 41.66 |
| GIV/HM543471 | 26.86 | 41.5 |
| BTV/L11723 | 23.41 | 44.3 |
| BTV(W)/GQ506542 | 23.99 | 43.17 |
| TOV/EU839843 | 21.97 | 43.66 |
| EHDV2(W)/AM745003 | 22.83 | 44.32 |
| EHDV2(E)/AM744993 | 22.25 | 43.51 |
| AHSV/HM035361 | 23.41 | 43.34 |
| CHUV/NC005988 | 26.38 | 41.86 |
| EEV/FJ183391 | 24.21 | 40.2 |
| SCRV/NC_006004 | 16.07 | 36.9 |
